# Biomimetic Mineralization of Magnetic Iron Oxide Nanoparticles Mediated by Bi-Functional Copolypeptides

**DOI:** 10.3390/molecules24071401

**Published:** 2019-04-10

**Authors:** Liu Liu, Ximing Pu, Guangfu Yin, Xianchun Chen, Jie Yin, Yuhao Wu

**Affiliations:** 1 College of Materials Science and Engineering, Sichuan University, Chengdu 610065, China; liuliu2019@126.com (L.L.); yingf@scu.edu.cn (G.Y.); kfwcxc@163.com (X.C.); inj21@163.com (J.Y.); 15198006630@163.com (Y.W.); 2 School of Automation and Information Engineering, Sichuan University of Science and Engineering, Zigong 643000, China

**Keywords:** Fe_3_O_4_ nanoparticle, biomimetic mineralization, A2780 cell, targeting

## Abstract

Magnetite (Fe_3_O_4_) nanoparticles are widely used in multiple biomedical applications due to their magnetic properties depending on the size, shape and organization of the crystals. However, the crystal growth and morphology of Fe_3_O_4_ nanoparticles remain difficult to control without using organic solvent or a high temperature. Inspired by the natural biomineralization process, a 14-mer bi-functional copolypeptide, leveraging the affinity of binding Fe_3_O_4_ together with targeting ovarian cancer cell A2780, was used as a template in the biomimetic mineralization of magnetite. Alongside this, a ginger extract was applied as an antioxidant and a size-conditioning agent of Fe_3_O_4_ crystals. As a result of the cooperative effects of the peptide and the ginger extract, the size and dispersibility of Fe_3_O_4_ were controlled based on the interaction of the amino acid and the ginger extract. Our study also demonstrated that the obtained particles with superparamagnetism could selectively be taken up by A2780 cells. In summary, the Fe_3_O_4_-QY-G nanoparticles may have potential applications in targeting tumor therapy or angiography.

## 1. Introduction

Fe_3_O_4_ magnetic nanoparticles (Fe_3_O_4_-MNPs) have gradually become the concern of researchers because they can be applied in the fields of labeling, magnetic separation of biological materials, Magnetic Resonance Imaging (MRI) contrast enhancement, directed drug delivery, and hyperthermia treatment [1,2]. The properties and applications of Fe_3_O_4_-MNPs depend largely on size, crystallinity, and morphology. Fe_3_O_4_-MNPs could display a unique form of magnetism called superparamagnetism [3] when the diameter is below a certain size (generally 25–30 nm), which makes them respond more rapidly and stronger than the bulk magnets in a magnetic field [4]. The nanoparticles retain no residual magnetism at room temperature with negligible coercivity, therefore, these particles could easily be dispersed when the magnetic field is removed, which is conducive to increasing the half-life of the particles in the circulation through escaping from macrophages.

However, the superparamagnetic Fe_3_O_4_-MNPs are easily oxidized in an aqueous solution, which would lead to reuniting. The aggregates of magnetic particles will be removed by phagocytes, or they may cause thrombosis or the blockage of blood capillaries after entering organisms. In addition to this, it is inevitable that some Fe_3_O_4_-MNPs obtained by one-pot method contain large amounts of organic solvents, which will limit the applications of the materials in organisms. Therefore, how to prepare Fe_3_O_4_-MNPs with suitable size and excellent dispersibility through a safe and convenient method has become a research hotspot in the biomedical field. 

Biomimetic mineralization is a powerful approach to synthesize advanced materials with controlled size, shape, and polymorph under ambient conditions and pressure in an aqueous environment, using biological macromolecules (peptide, protein, DNA, etc.) as templates [5,6,7]. Many proteins/peptides have been well developed for metal nanoparticle (NP) biosynthesis, such as Au NPs [8,9] and Gd NPs [10], along with the associated synthetic theories. As well as this, there were studies conducted on the biosynthesis of Fe_3_O_4_ or Fe_2_O_3_. For example, Mms6 [11], a magnetosome-associated protein derived from Magnetospirillum magneticum strain AMB-1, regulated Fe_3_O_4_ crystal morphology and promoted the formation of superparamagnetic Fe_3_O_4_-MNPs of uniform size and shape. Also, block copolypeptides poly(EG_2_-lys)_100_-b-poly(asp)_30_ [12] were used as a coating to stabilize clusters of Fe_2_O_3_-MNPs and control their sizes in an aqueous environment. Therefore, it is speculated that functional biological molecules (e.g., targeting peptide [13], polypeptide with a therapeutic effect [14], protein, or biopolymer capable of improving biocompatibility [15]) can be introduced in the process of biomimetic mineralization so that the specific functions can be endowed at the same time as Fe_3_O_4_-MNPs are being formed.

Green chemistry synthesis methods for nanoparticles have positive aspects compared with chemical methods, such as safety, environment amity, and no use of harmful compounds [16]. Plant extracts are generally considered reducing and stabilizing agents for the synthesis of metal nanoparticles with specific shapes and sizes [17,18,19]. The phytochemicals including hydroxyl, carboxyl, and amino functional groups can be regarded as effective metal-reducing agents. They can both improve the stability of nanoparticles and give rise to a cooperative effect when the nanoparticles are used for biomedical applications. Among them, ginger extract has attracted attention because of its chemical and biological properties such as antioxidant, antibacterial activity, and anti-inflammatory effects [20]. Ginger is a common condiment for various foods and beverages. As a functional food, ginger has been recognized for its health benefits [21]. It has long history of medical use in many countries. Besides minerals such as iron, calcium, phosphorous, zinc, copper, chromium and manganese, ginger also contains dietary fiber and sugar, lipids vitamins, and amino acids. The components are harmless to the human body. The microstructure of ginger extract has also been confirmed to increase the stability of synthetic material [22].

In this work, Fe_3_O_4_ nanoparticles (Fe_3_O_4_-QY-G) with good targeting performance and morphology were prepared by biomimetic mineralization in a mild condition using 14-mer peptide (QQTNWSLTVNFKLY, QY) as templates together with the ginger extract. This 14-mer peptide was designed in a predetermined manner, which consisted of a peptide (TVNFKLY, TVN) with Fe_3_O_4_-MNPs affinity [23] and a peptide (QQTNWSL, QQT) which targets the ovarian carcinoma cell A2780. These 7-mer peptides were screened previously using phage display peptide library technology.

## 2. Results

### 2.1. Characterization of Mineral Phases

**Figure 1:**
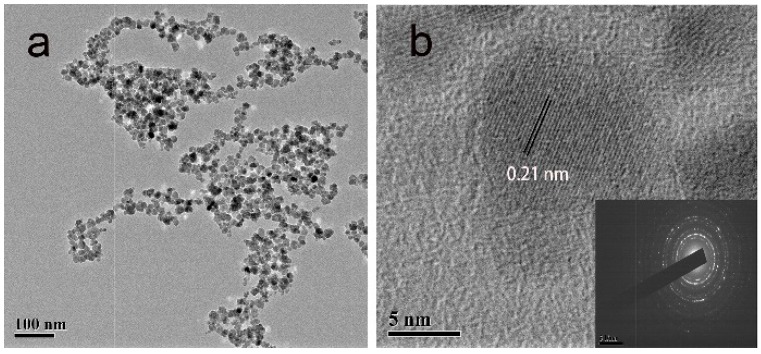
(**a**) Transmission electron microscopy (TEM) and (**b**) high-resolution transmission electron microscopy (HR TEM) images of Fe_3_O_4_ nanoparticles prepared with TVNFKLY (TVN) peptide.

Firstly, to verify that peptide TVNFKLY (TVN) could mediate the formation of Fe_3_O_4_, Fe_3_O_4_-peptide nanoparticles were prepared using the method described in 4.2 with different concentrations of TVN and a random peptide named SVE (the specific data is shown in the supporting information, Figure S1). As shown in Figure 1, the Fe_3_O_4_-TVN nanoparticles presented good dispersibility and uniform size (details are presented in the supporting information, Figure S1b). The TVN peptide was combined with an ovarian carcinoma cell A2780 specific targeting peptide (QQT) to construct a 14-mer bi-functional copolypeptide (QY) which was expected to possess both the affinity of binding Fe_3_O_4_ and targeting the ovarian cancer cell A2780. In the following sections, the Fe_3_O_4_ nanoparticles prepared by simultaneously using the QY peptide and ginger extract are referred to as Fe_3_O_4_-QY-G, while the Fe_3_O_4_ nanoparticles prepared by using the QY peptide are referred to as Fe_3_O_4_-QY (the relevant data on concentration investigation is shown in the supporting information, Figure S4).

**Figure 2:**
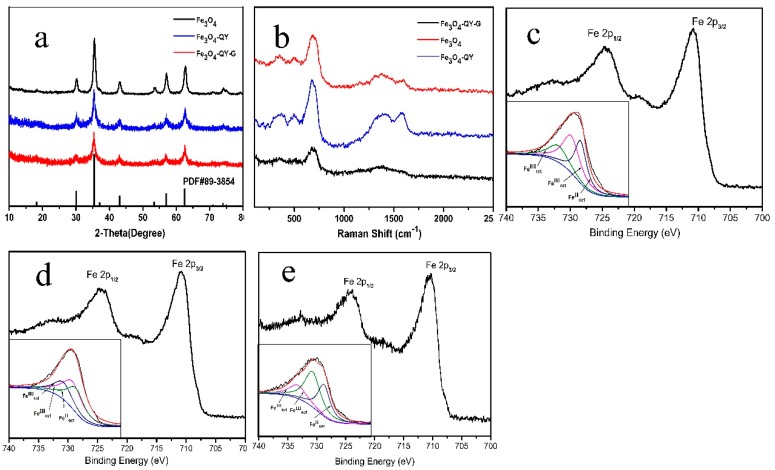
(**a**) XRD pattern of Fe_3_O_4_, Fe_3_O_4_-QY, Fe_3_O_4_-QY-G nanoparticles, (**b**) Raman spectrum of Fe_3_O_4_, Fe_3_O_4_-QY, Fe_3_O_4_-QY-G nanoparticles, XPS Fe 2p core-level spectra of (**c**) Fe_3_O_4_, (**d**) Fe_3_O_4_-QY, (**e**) Fe_3_O_4_-QY-G.

The results of X-ray diffraction (XRD), X-ray photoelectron spectroscop (XPS) and Raman spectroscopy showed that all the Fe_3_O_4_ nanoparticles were cubic inverse spinel structure. This means that the addition of the peptide and ginger extract have no significant influence on the crystal structure of Fe_3_O_4_. The XRD pattern of Fe_3_O_4_-QY-G nanoparticles is shown in Figure 2a. The characteristic peaks at 30.1°, 35.69°, 43.24°, 57.13°, and 62.77° were corresponding to the (220), (311), (400), (511), and (440) crystal planes of Fe_3_O_4_ (Joint Committee on Powder Diffraction Standards (JCPDS) No.89-3854), respectively. With the XRD pattern, the average diameter which could be evaluated from the Scherrer equation (D = *Kλ*/*β*cos*θ*, where *K* is a constant (0.89), *λ* is the X-ray wavelength (1.54060 Å), *β* is the peak width of half-maximum, and *θ* is the Bragg diffraction angle) and was obtained as 6–12 nm. In addition, the broad peak in the 5–23° range was speculated to be organic compounds from the ginger extract [24]. The peaks shown at 517 cm^−1^ and 680 cm^−1^ in Figure 2b were typical Raman scattering peaks of Fe_3_O_4_ [25,26] and the peaks at 1400 cm^−1^ and 1580 cm^−1^ were the characteristic peaks of γ-Fe_2_O_3_ [27], which revealed that Fe_3_O_4_ was oxidized to Fe_2_O_3_ under the heating condition [28]. From Figure 2c–e, it can be seen that the peak positions of Fe 2p_3/2_ and Fe 2p_1/2_ are located at around 710.34 eV and 724.11 eV, respectively. The Fe 2p_3/2_ peak can be well fitted by peaks of octahedral ferrous iron [Fe (II) oct.], octahedral ferric iron [Fe (III) oct.], and tetrahedral ferric iron [Fe (III) tet.]. The Fe (II)/Fe (III) ratio of Fe_3_O_4_ (Figure 2c), Fe_3_O_4_-QY nanoparticles (Figure 2d), and Fe_3_O_4_-QY-G nanoparticles (Figure 2e) were 0.503, 0.498 and 0.501, respectively. All of these matched the theoretical value of Fe_3_O_4_. Moreover, no shakeup satellite peaks, which are the fingerprints of the electronic structures of iron oxides such as α-Fe_2_O_3_ and γ-Fe_2_O_3_, can be identified. This indicates that Fe_2_O_3_ does not present in the nanoparticles.

### 2.2. Constituent Analysis via FT-IR

**Figure 3:**
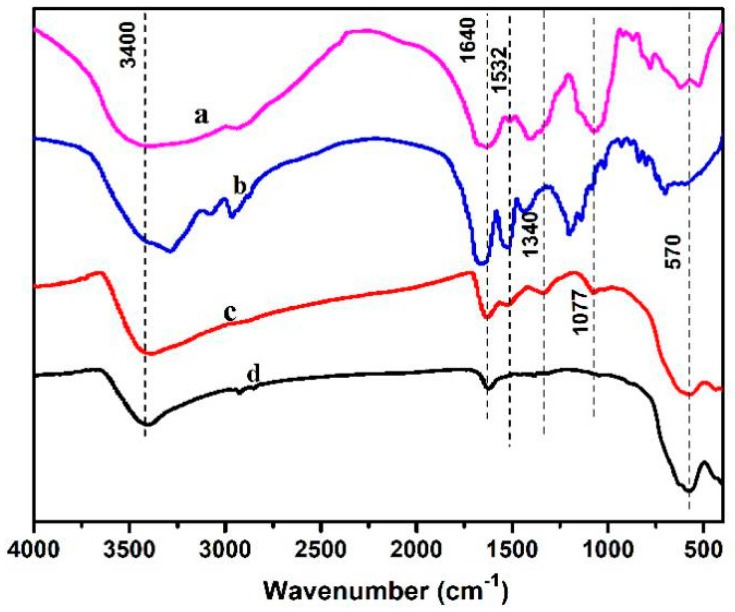
FT-IR spectra of (**a**) ginger extract, (**b**) peptide (QY), (**c**) Fe_3_O_4_-QY-G nanoparticles, (**d**) Fe_3_O_4_ nanoparticles.

Figure 3 shows an FT-IR image of Fe_3_O_4_ nanoparticles, 14-mer peptide, ginger extract, and Fe_3_O_4_-QY-G nanoparticles. Ginger contains a complex chemical composition: In addition to dietary fiber and sugar, it also contains specific compounds—gingerol (C_17_H_26_O_4_), minerals, flavonoids, lipids, vitamins, and amino acids [29,30]. Complex peaks in the range of 400–800 cm^−1^ may be mineral compounds, most of which contain K, Mg, P, Ca, and Na elements [31]. The peaks at 1320–1420 cm^−1^ were related to O–H bending vibrations of the phenolic group and C–H bending vibrations (saccharides, gingerol, flavonoids, and vitamins). The peak at 1077 cm^−1^ may be prescribed to C=O stretching vibrations of organic compounds (mainly saccharides) [32]. The strong broad peaks centered at 3400 cm^−1^ were ascribed to the stretching vibrations of H-bonded O–H groups. The peaks at 1640 cm^−1^ were related to H_2_O bending vibrations. The comparison of peak intensity showed that the ginger extract was mainly composed from saccharides (including starch) with admixtures of mineral components and phenolic compounds. With respect to the peptide, the amide I band results from the stretching vibration of the peptide carbonyl group (C=O); the native spectrum of this band shows three components positioned at 1633 cm^−1^, 1643 cm^−1^, and 1660 cm^−1^ [22]. 

For the Fe_3_O_4_-QY-G nanoparticles, the band at 1532 cm^−1^ was attributed to amide II. The peak at 1077 cm^−1^ may be prescribed to the ginger extract. The broad peak at 570 cm^−1^ belonged to stretching vibrations of Fe–O in Fe_3_O_4_.

The difference in peak intensity among the ginger extract, peptide, and Fe_3_O_4_ nanoparticles points to the selective adsorption of the ginger extract and peptide on the nanoparticle surface. Aside from this, the peaks at 1660 cm^−1^ and 1433 cm^−1^ which were related to the amide I and the amide III, respectively, disappeared in the Fe_3_O_4_-QY-G nanoparticles. The peak at 1410 cm^−1^ shifted to 1340 cm^−1^ (C–H bending vibration). The disappearance of amide bonds may have been caused by C=O forming a hydrogen bond with the -OH enriched on the surface of Fe_3_O_4_. Specifically, the iron atoms in the surface of the Fe_3_O_4_ atomic lattice may coordinate with water molecules in an aqueous solution and form -OH, which could form hydrogen bonds with the C=O from amide. Meanwhile, the peak at 3400 cm^−1^ of the Fe_3_O_4_-QY-G nanoparticles had a larger intensity than that of the Fe_3_O_4_ nanoparticles, which indicated an increased hydrophilicity of the prepared material.

### 2.3. Morphology and Solvent-Dependent Stability 

**Figure 4:**
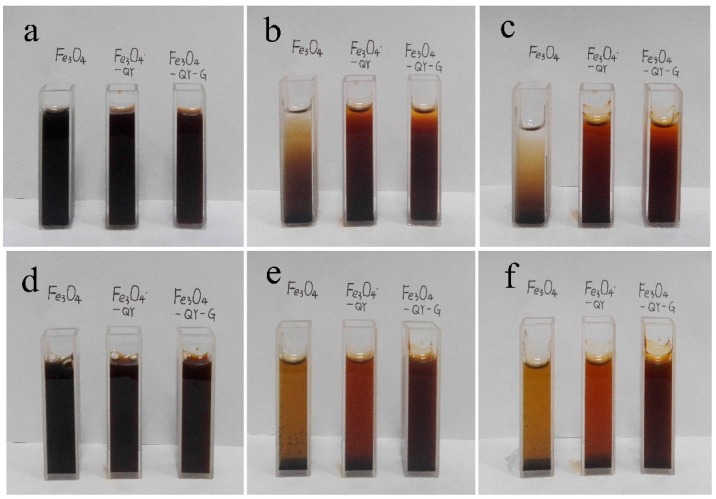
Solvent-dependent stability pictures of nanoparticles (**a**) 0 h in water, (**b**) 24 h in water, (**c**) 48 h in water, (**d**) 0 h in human serum, (**e**) 24 h in human serum, (**f**) 48 h in human serum.

To observe the stability of the different materials, water and human serum were used as dispersants. As shown in Figure 4a–f, after incubation for 48 h, the Fe_3_O_4_ nanoparticles completely settled in the aqueous phase while Fe_3_O_4_-QY-G nanoparticles were stable with only a slight subsidence. As a dispersion medium, the serum would reduce the sedimentation rate of nanoparticles because of its viscosity. In addition to this, the residual phenolic groups contained in the ginger extract may protect Fe_3_O_4_ from oxidization and slow down the aggregation and sedimentation rate. As demonstrated above, the Fe_3_O_4_-QY-G nanoparticles are more stable than Fe_3_O_4_ nanoparticles in the same condition.

Additionally, the lysine (K) and asparagine (N) side chains in the 14-mer peptide can spontaneously form chemically stable amide bonds, namely isopeptide bonds [33,34,35]. The isopeptide bonds decrease the effect of hydrolytic degradation, and increase the stability of the peptide linkage and the affinity for the targeting ligand, which is favorable for the subsequent application of the material. Since Fe_3_O_4_-QY-G nanoparticles show greater stability than the Fe_3_O_4_-QY nanoparticles, the subsequent characterizations mainly focus on Fe_3_O_4_-QY-G.

**Figure 5:**
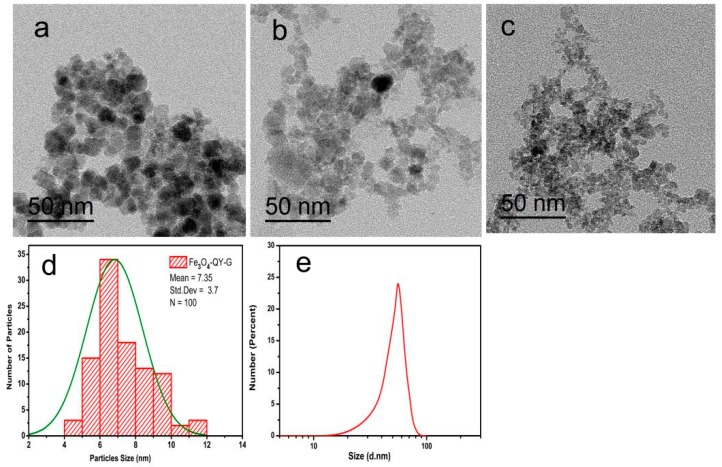
TEM images of nanoparticles: (**a**) Fe_3_O_4_, (**b**) Fe_3_O_4_-QY, (**c**) Fe_3_O_4_-QY-G, (**d**) corresponding size distribution of Fe_3_O_4_-QY-G nanoparticles, (**e**) hydrodynamic diameter of Fe_3_O_4_-QY-G nanoparticles.

TEM observation was used to examine the difference in particle size among the samples. The TEM image shows that Fe_3_O_4_-QY-G nanoparticles (Figure 5c) were of a small size. However, glomeration occurred because the extremely small particle size can access greater specific surface area and surface energy. The average particle size was about 7 nm, and the Fe_3_O_4_-QY nanoparticles (Figure 5b) showed a diameter of around 10 nm. While under the same condition, the Fe_3_O_4_ nanoparticles (Figure 5a) prepared by the coprecipitation method had a diameter of about 15 nm, and aggregated severely. 

As shown in Figure 5c, the particle size distribution curve of Fe_3_O_4_-QY-G indicated that the mean diameter size of the nanoparticles was 7.35 ± 3.7 nm. The XRD pattern suggested that the unassigned peaks may indicate the crystallization of the bio-organic phase present in the extract, which was also observed from the TEM image. The good correlation between particle sizes obtained from the Scherrer equation and TEM supports the crystalline structure of the iron nanoparticles. Zeta-potential and dynamic light scattering (DLS) are additional characterization methods to further determine the dispersion and stability of the Fe_3_O_4_-QY-G nanoparticles. DLS measurement (Figure 5e) indicated that these samples render suspension with a mean hydrodynamic diameter of about 55.28 nm due to the presence of associated and hydrated organic layers [36]. As DLS is weighted towards large sizes, the average diameter could be higher than those obtained from TEM. However, the average hydrodynamic diameter and the feature that Polydispersity Index (PDI) = 0.102 hardly varied with time could reveal the excellent stability of Fe_3_O_4_-QY-G nanoparticles. In addition to this, the highly negative value of zeta-potential of these functionalized nanoparticles in water (ζ = −18.9 mV) also indicated good stability. When the particles are stabilized by purely electrostatic repulsion, an absolute zeta potential value of more than 25 mV is ideal for good kinetic stability at room temperature. However, in the presence of other forces such as steric and hydrogen bonding interactions, the stability can be achieved even at low surface potentials [37]. 

### 2.4. Magnetic Property and Imaging Efficiency

**Figure 6:**
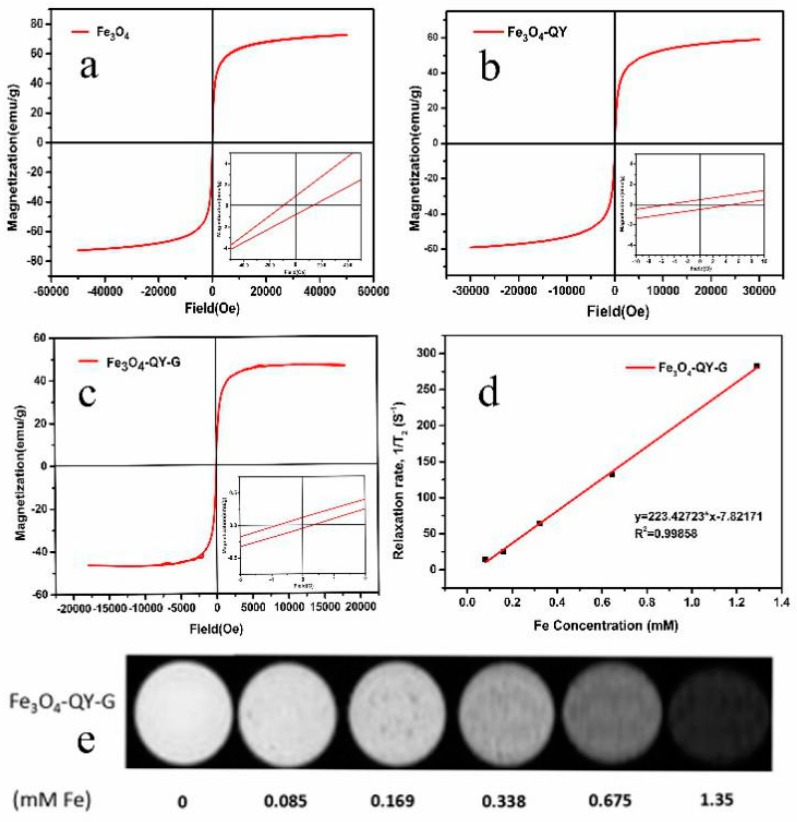
Magnetization curves of (**a**) Fe_3_O_4_, (**b**) Fe_3_O_4_-QY, (**c**) Fe_3_O_4_-QY-G nanoparticles; (**d**) T_2_ relaxation rates as a function of iron concentration, (**e**) T_2_-weighted Magnetic Resonance (MR) images of Fe_3_O_4_-QY-G nanoparticles with different Fe concentration.

The magnetization curves of Fe_3_O_4_, Fe_3_O_4_-QY, and Fe_3_O_4_-QY-G nanoparticles obtained by Vibrating Sample Magnetometer (VSM) at room temperature are shown in Figure 6a–c. The Fe_3_O_4_-QY and Fe_3_O_4_-QY-G nanoparticles exhibited the characteristics of superparamagnetism. The coercivity (Hc) and saturation magnetization (Ms) of Fe_3_O_4_-QY-G nanoparticles were 0.35 Oe and 48.9 emu/g, respectively (Figure 6c). As for the Fe_3_O_4_-QY nanoparticles, the Ms and Hc were 58.2 emu/g and 4.52 Oe, respectively (Figure 6b). Both of these Ms values were lower than that of Fe_3_O_4_ nanoparticles prepared by coprecipitation (72.5 emu/g, Figure 6a). In addition to the adsorption of organic compounds and peptide on nanoparticles (including physical and chemical adsorption), the decrease of saturation magnetization may also be attributable to the size and surface effect. For particles smaller than 10 nm, saturation magnetization is smaller, most likely due to the surface spin canting of the small magnetic nanoparticles.

Since superparamagnetic Fe_3_O_4_-MNPs are highly sensitive to external magnetic fields, they are widely used as a contrast agent. Superparamagnetic nanoparticles with strong magnetic moments can locally amplify the external magnetic field to make it non-uniform and shorten the T_2_ relaxation time of the tissue. Figure 6 shows (**d**) T_2_ relaxation rates as a function of iron concentration, and (**e**) a series of T_2_-weighted MR images of Fe_3_O_4_-QY-G nanoparticles with different Fe concentration. Taking the slope of a linear fit of 1/T_2_ vs. Fe concentration, the transverse relaxivity (r_2_) value of Fe_3_O_4_-QY-G in 5% agarose was 223 mM^−1^S^−1^, which was two times higher than that of Feraheme (ferumoxytol, iron oxide nanoparticles with an r_2_ value of 98.4 mM^−1^S^−1^ at 7 T). Therefore, the Fe_3_O_4_-QY-G nanoparticles have great potential in the application of contrast agents.

### 2.5. The Chelating Ability and Reduction Capability Results of Ginger Extract

**Figure 7:**
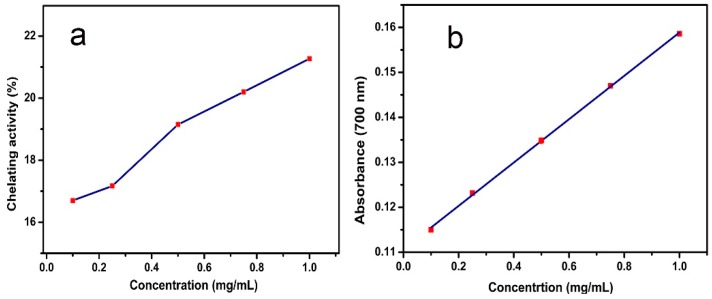
(**a**) The chelating ability on Fe^2+^ of ginger extract; (**b**) the reduction capability of Fe^3+^ of ginger extract.

Chen and Ahn [38] found that natural phenolics including quercetin, rutin, catechin, and caffeic acid could be used as a Fe^2+^-chelator. The data revealed that ginger extract could also function as a Fe^2+^-chelator because of its natural phenols. Figure 7a shows that the chelating activity of ginger extract on Fe^2+^ is concentration-dependent. When a small amount of ginger extract (0.1–2 mg/mL) was added to the reaction system, it could suppress reactivity by occupying the coordination sites of metal ions due to its ferrous ion chelation.

In addition to this, as is well-known, the oxidation resistance mechanism of phenolic antioxidants is based on donating an electron to a free radical from OH- groups attached to the phenolic rings. The mechanism of gingerol antioxidant activity is considered to be similar to that of piperine and curcumin. It is correlated to the phenolic OH- group and the CH_2_- group of the β-diketone moiety. The free radical can undergo electron transfer or abstract an H- atom from either of these two sites of gingerol molecule. The ability of the extract to reduce iron (III) was assessed by the method of Oyaizu [39]. In this method, antioxidants form a colored complex with K_3_[Fe(CN)_6_] in the presence of trichloroacetic acid and ferric chloride. Measured at 700 nm, an increase in the absorbance of the reaction mixture indicates the reducing power of the sample. At lower concentrations (0.1–2 mg/mL), the reducing ability of ginger extract to Fe^3+^ was correlated with its concentration. Along with an increase in extract concentration, the ability to reduce the Fe^3+^ is also strengthened (Figure 7b).

### 2.6. Cell Targeting and Cytotoxicity Analysis 

**Figure 8:**
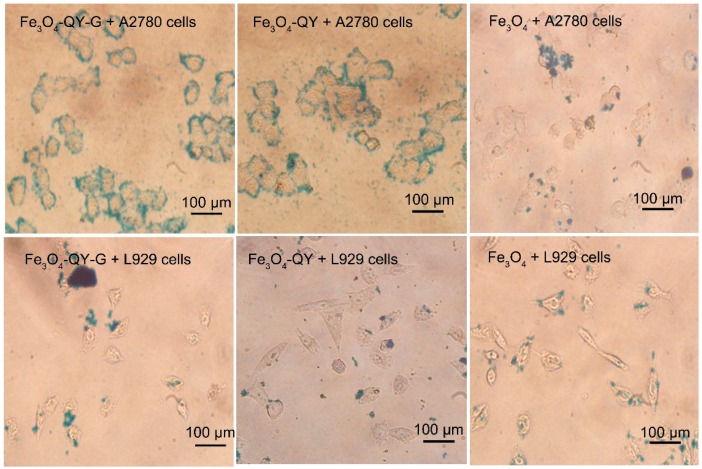
Prussian blue staining images of A2780 and L929 cells incubated with Fe_3_O_4_-QY-G, Fe_3_O_4_-QY, or Fe_3_O_4_ nanoparticles for 4 h.

The targeting ability of Fe_3_O_4_-QY and Fe_3_O_4_-QY-G nanoparticles to tumor cells was preliminarily examined through Prussian blue staining. In Figure 8, a strong adsorption of Fe_3_O_4_-QY and Fe_3_O_4_-QY-G nanoparticles was observed in A2780 cells, while there was only a slight adsorption of Fe_3_O_4_ nanoparticles in the same condition. Moreover, the L929 cells were used as a control, and there was no significant adsorption of nanoparticles. The results above preliminarily proved that the Fe_3_O_4_-QY and Fe_3_O_4_-QY-G nanoparticles have targeting behavior to A2780 cells due to the peptide (QY) and the addition of ginger extract did not significantly affect the targeting ability of the materials. 

**Figure 9:**
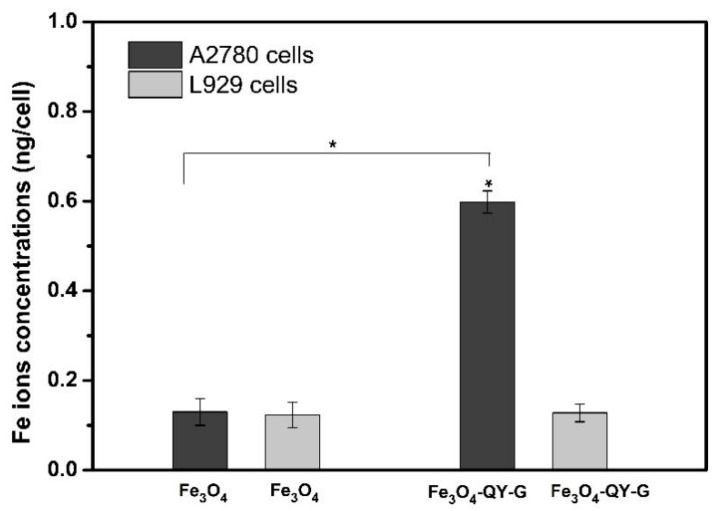
The Fe ion concentration in each A2780 and L929 cell after co-cultured with Fe_3_O_4_-QY-G and Fe_3_O_4_ nanoparticles for 24 h at a concentration of 100 µg/mL in the medium, * shows significant differences between the corresponding groups, * *p* < 0.05.

To further study the targeting specificity of the Fe_3_O_4_-QY-G nanoparticles, the nanoparticles were incubated with A2780 cells and L929 cells. Figure 9 shows the Fe uptake of Fe_3_O_4_-QY-G nanoparticles by A2780 cells, which is about 4.86-fold higher that by L929 cells. As in the group of Fe_3_O_4_ nanoparticles, no significant difference was observed for iron uptake in either A2780 cells or L929 cells. The quantitative results by Inductively Coupled Plasma Optical Emission Spectrometer (ICP-OES) analysis are consistent with the qualitative data by microscope. Thus, the obtained Fe_3_O_4_-QY-G nanoparticles are anticipated to have great potential for use in the field of cancer diagnosis and treatment. 

**Figure 10:**
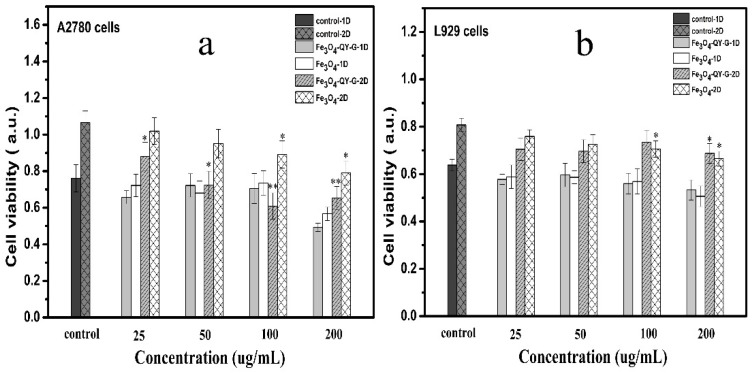
Effect of Fe_3_O_4_-QY-G and Fe_3_O_4_ nanoparticles with different concentrations on the viability of (**a**) A2780 cells, (**b**) L929 cells. (* and ** show significant differences between groups treated with control and nanoparticles. * *p* < 0.05, ** *p* < 0.01. Data are presented as means ± SD, *n* = 6).

Figure 10 shows the cell viability after incubation with different concentrations of Fe_3_O_4_-QY-G nanoparticles and Fe_3_O_4_ nanoparticles. It can be seen that the cell viability of A2780 cells was not significantly affected at the concentrations of 25–100 µg/mL after 24 h. While the concentration increased to 200 µg/mL, both the two materials showed a little cytotoxicity. When the co-culture time reached 48 h, the cells exposed to the Fe_3_O_4_-QY-G nanoparticles were fewer than those exposed to the Fe_3_O_4_ nanoparticles and the control. These results indicated that the Fe_3_O_4_-QY-G nanoparticles could inhibit A2780 cell proliferation. With regard to the L929 cells (Figure 10b), there was no significant cytotoxicity seen in both groups of Fe_3_O_4_-QY-G nanoparticles and Fe_3_O_4_ nanoparticles. 

## 3. Discussion

The newly developed Fe_3_O_4_-QY-G nanoparticles in this study showed high r_2_ relaxivity and targeting ability. Unlike the Fe_3_O_4_ nanoparticles prepared in a one-pot strategy reported previously, these Fe_3_O_4_-QY-G nanoparticles possess a small particle size, superparamagnetism, and good dispersion stability. The presence of the additives can enhance, retard, or inhibit crystal nucleation, and therefore assist in the formation of a desired crystal habit. The key factors that determine the ability of an additive to modulate crystal nucleation are the strength of its interaction with the solute, its disruptive ability, interfacial properties, and the degree of self-association [40]. For additives with a high affinity for the solute, the solute molecules tend to conflict with the emerging solute lattice and hence cause nucleation inhibition. Since the shape and size of the inorganic particles are correlative to the formation process of crystal in biomimetic mineralization, we should bear these factors in mind when discussing the advance of this method.

Firstly, the overall physicochemical properties (e.g., charge density and hydrophilicity) of the amino acid active domain were speculated to be responsible for their impact in the biomineralization process [41,42,43], based on the premise that amino acid residue with hydrophilic side chains could interact with the lattice structure of the inorganic material. According to the peptide side chain groups shown in Table S1 (Supporting Information), the hydrophilic amino acids: glutamine (Q), threonine (T), asparagine (N), serine (S), lysine (K), and tyrosine (Y) account for 64.3% and the polar amino acids: Q, T, S, K, and Y account for 42.9%, which indicated that the 14-mer peptide possessing hydrophilic side chains could interact with the lattice structure of Fe_3_O_4_ nanoparticles [44]. 

**Figure 11:**
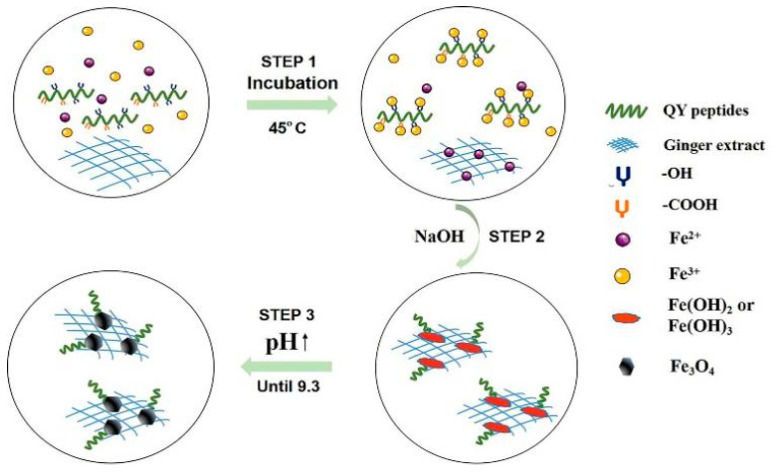
Possible formation mechanism of Fe_3_O_4_-QY-G nanoparticles.

Secondly, the peptide worked synergistically with the ginger extract to actively control the biomimetic mineralization as a Fe_3_O_4_ nucleation inhibitor (Figure 11). In this system, hydroxyl-containing amino acids (T, Y, and S) and carboxyl-containing amino acids (Q) can be regarded as hard Lewis bases and react with Fe^3+^, which was considered as a hard acid by the electrostatic interaction between the hard acid and the hard base [18]. Besides this, the chelating ability on Fe^2+^ of the ginger extract limited the migration of ions and caused them to disperse homogeneously (Figure 11, Step 1). This could make up for the fact that normally mineralized proteins only bind and disperse Fe^3+^. Another active ingredient in the process may be the polysaccharide (e.g., starch). The existence of the hydrogen bonds between the functional groups of polysaccharides and the oxygen atoms of the Fe_3_O_4_ (111) surface has been proven by the radial distribution function analysis [45], which may be the reason for the forming of a strong interaction. However, the appropriate reaction time and additive concentration did not significantly change the growth rate of different crystal facets, and the prepared nanoparticles remained spherical in this study.

**Figure 12:**
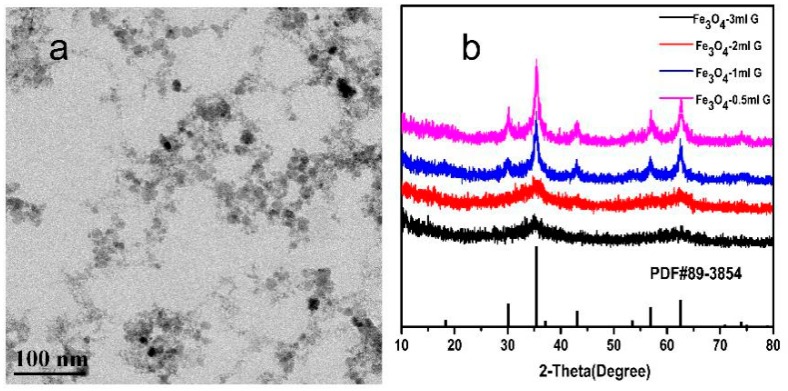
(**a**) TEM image of Fe_3_O_4_ nanoparticles prepared by adding 3 mL ginger extract, (**b**) XRD pattern of Fe_3_O_4_ nanoparticles prepared by adding different concentrations of ginger extract.

Based on the analysis presented above, the peptide and ginger extract performed as nucleation inhibitors by binding to the precursors, and would postpone the conversion of precursors to Fe_3_O_4_. As a consequence, Fe_3_O_4_ would be harvested at a higher pH condition due to the combination mentioned above, and the smaller crystal size would be produced because of the decrease in surface tension [46,47,48] (Figure 11, Step 2). Also, nucleation of iron oxide particulates occurs relatively free from interparticle interactions (e.g., aggregation) as a result of the templating action. Furthermore, the microstructure of the ginger extract is mainly composed of lamellaes and nets. The former consists of a dense alternating layer with a spacing of 3–5 µm, whereas the latter consists of fibers less than 100 nm in diameter [22]. Therefore, in the microenvironment, the lamellaes and nets of ginger may limit the growth and aggregation space of the Fe_3_O_4_ crystal (Figure 11, Step 3). However, the more ginger extract was added, the lower the crystallinity was. When the amount reached 3 mL (containing 60 mg of ginger extract powder), the crystallization could hardly be observed (Figure 12, Figure S2). This may be due to the chelating ability and reduction capability of the ginger extract, causing complete inhibition of crystallization.

Finally, with the protonation/deprotonation of K, the positively charged residues in the peptide could control the dispersion/precipitation of the particles, which affected the particle dispersion [49]. Under alkaline conditions of the reaction system (pH = 9.3), the increase in electrostatic stabilization upon protonation of the K residues protects the particles against uncontrolled agglomeration. Meanwhile, the iron atoms in the surface of the Fe_3_O_4_ atomic lattice coordinated with water molecules in an aqueous solution, leading to the enrichment of hydroxyl groups (-OH) on the surface of particles, and the binding of C=O contained in the peptide to the hydroxyl terminated Fe_3_O_4_ surface via hydrogen bonds [50]. Hence, the presence of hydrogen bonds prolonged the protonation of K and improved the dispersibility of particles [51]. 

In summary, superparamagnetic and functionalized Fe_3_O_4_ nanoparticles were obtained under the mediation of a 14-mer peptide and ginger extract.

## 4. Materials and Methods 

### 4.1. Materials 

FeCl_2_·4H_2_O, FeCl_3_·6H_2_O, and NaOH were obtained from the Chengdu Kelong Chemical Reagent Factory (Chengdu, China). The 14-mer peptide (QQTNWSLTVNFKLY), synthesized by Shanghai Bootech BioScience and Technology Co., Ltd., was applied as a template. The ginger was bought in the local market (Chengdu, China). All reagents were of analytical grade and used directly without further purification, and aqueous solutions were used with deionized water (18.25 MΩ·cm resistivity at 25 °C). L929 cells and A2780 cells were purchased from the Shanghai Institute of Biochemistry and Cell Biology (Shanghai, China), and Fenghui Biotechnologies Inc. (Changsha, China), respectively.

To prepare the ginger extract, the ginger (250 g) was cleaned and washed with distilled water and then cut into small pieces. The extraction of constituents was carried out using a water–ethanol solution (300 mL, 1:1 ratio) for 5 days at room temperature (in a dark location). Then the supernatant was vacuum filtered and freeze-dried to obtain the powder. The ginger extract solution (20 mg/mL) was prepared fresh and used right after it was ready. 

### 4.2. Preparation of Fe_3_O_4_ Nanoparticles 

14-mer peptide (30, 40, 50, and 60 mg) was dissolved in a solution (50 mL) that was ferric and ferrous (molar ratio = 2/1) with a certain amount of ginger extract (0.5, 1, 2 and 3 mL). After interacting for 30 min at 45 °C and in a N_2_ atmosphere, an appropriate volume of NaOH (25%) was dropwise added with dripping rate of 1–2 drop/s until the pH approximately reached 9.3. The reaction under this condition lasted for 5 h, and following this the suspension was centrifuged. Then, the precipitates were washed with deoxygenated distilled water and absolute ethyl alcohol until the pH of washed water was at about 7, and were then dried in a vacuum atmosphere. As the control, the Fe_3_O_4_-QY nanoparticles were prepared in the system without the ginger extract. Iron oxide of Fe^2+^ and Fe^3+^ were precipitated at an alkaline pH to get the desired Fe_3_O_4_ MNPs as shown below:

Fe^2+^ + 2Fe^3+^ + 8OH^−^ = Fe_3_O_4_↓ + 4H_2_O

### 4.3. Physicochemical Characterizations

The phase composition was collected via XRD analysis using Cu-Kα radiation (λ = 1.54060 Å) over the 2θ range of 5–80° at a rate of 5°/min with a Lab XRD-6100 diffractometer (Kyoto, Japan). The Raman scattering measurement was conducted at room temperature, and the excitation light was the 532 nm line. X-ray photoelectron spectroscopy (XPS, Escalab250Xi, Waltham, MA, USA) measurement was carried out to further verify the chemical composition. Transmission Electron Microscopy (Libra 200 FE, Oberkochen, Germany, operating at 200 kV) was used for characterizing the size and morphology of nanoparticles. The magnetic property was measured with a vibrating sample magnetometer (Lake shore-7400, Columbus, OH, USA) at room temperature. Thermal decomposition of the compound was studied from room temperature to 1000 °C by differential scanning calorimetry (DSC) and thermogravimetric (TG) analysis at a heating rate of 10 °C/min in a dynamic argon atmosphere (STA449F3, Bavaria, Germany) (Supporting Information, Figure S3). FT-IR analysis was performed using the KBr method (Nicolet 6700, Waltham, MA, USA). The transverse relaxation times (T_2_) and imaging ability of Fe_3_O_4_-QY-G nanoparticles were measured for different concentrations of Fe using a 7 T MRI scanner (Bruker BioSpec 70/30, Billerica, MA, USA). Dynamic light scattering (DLS) measurement and zeta-potential measurements were performed using Zetasizer Nano ZS (Malvern, UK).

### 4.4. The Chelating Ability on Fe^2+^ and Reduction Capability Test of Fe^3+^

The chelating activity of the ginger extract on Fe^2+^ was carried out according to Juntachote’s report [52]. Briefly, 1 mL samples of different concentrations (0.1–2 mg/mL) were diluted with 3.7 mL deionized water. Then, 0.1 mL of FeCl_2_ (2 mM) and 0.2 mL ferrozine (5 mM) were added. Ferrozine can form a complex with free Fe^2+^ but not with Fe^2+^ bound by a chelating agent. After incubating for 10 min at room temperature, the absorbance of reaction mixtures was recorded at 562 nm in a Versa Max spectrophotometer (Bio-Rad, Hercules, CA, USA). The chelating activity was calculated according to the following expression equation:

(1) $$Chelatingactivity[\%]=1-\frac{Abs}{Abc}\times 100$$

where *Abs* and *Abc* are the absorbance of sample and the measurement at 562 nm, respectively. 

The reducing capability of the ginger extract was measured according to Iris Hinneburg [53]. 1mL samples of different concentrations (0.1–2 mg/mL) were incubated for 30 min at 50 °C with 2.5 mL phosphate buffer (0.2 M, pH 6.6) and 2.5 mL aqueous potassium hexacyanoferrate K_3_[Fe(CN)_6_] solution (1%, *w*/*v*). The reaction was terminated by adding 2.5 mL trichloroacetic acid (10%, *w*/*v*). Then, the mixture was centrifuged for 10 min at 3000 rpm. The supernatant (2.5 mL) was mixed with 2.5 mL distilled water and 0.5 mL ferric chloride (0.1%, *w*/*v*). The absorbance was measured at 700 nm in a Versa Max spectrophotometer.

### 4.5. In Vitro Cytotoxicity Study of Fe_3_O_4_-QY-G Nanoparticles

An MTT [3-(4,5-dimethyl-2-thiazolyl)-2,5-diphenyl-2Htetrazolium bromide] assay was performed to evaluate the cellular compatibility of Fe_3_O_4_-QY-G nanoparticles. Cells were cultured in a 96-well plate (approximately 1 × 10^4^ cells per well) with Dulbecco’s Modified Eagle Medium (DMEM) containing 10% neonatal calf serum (NCS) and different concentrations of the samples for 24 h and 48 h. Subsequently, 20 µL of MTT solution (5 mg/mL MTT in phosphate buffer solution) was added to each well and incubated for 4 h at 37 °C. After the medium was removed, 150 mL of dimethylsulfoxide (DMSO) was used to extract intracellular formazan crystals. The results were quantified by measuring the absorbance of the cell lysate at 490 nm. All results were averages ± SD of six samples. 

### 4.6. Cell Targeting Study of Fe_3_O_4_-QY-G Nanoparticles

Prussian blue staining was used to reveal the presence of iron cations. The A2780 and L929 cells were fixed with 4% formaldehyde after culturing with the Fe_3_O_4_-QY-G nanoparticles for 4 h and washed with phosphate buffer saline (PBS), followed by the incubation with 4 mL Prussian blue solution comprising an equal volume of 2% hydrochloric acid aqueous solution and 2% potassium ferrocyanide (II) trihydrate for 30 min. Finally, the iron staining was observed using a microscope (Olympus IX71, Japan) after being washed three times with PBS.

L929 and A2780 cells were cultured in a Petri dish (Φ = 100 mm) with a density of 4×10^5^ cells per Petri dish. After plating, the cells were washed three times with PBS, and the solution of nanoparticles was added with a concentration of 100 µg/mL. The cells were incubated for 24 h and washed three times with PBS to remove extra nanoparticles. After detaching by trypsin solution, the detached cells were counted and then prepared for ICP-OES (Agilent ICPOES730, Santa Clara, CA, USA). 

### 4.7. Statistical Analysis

Statistical comparisons were made by analysis of variance (ANOVA). The mean ± standard deviation was calculated in each experiment (*n* = 6), with statistical significance considered when *p* ≤ 0.05.

## 5. Conclusions

In this work, the 14-mer peptide capable of specific binding to both Fe_3_O_4_-MNPs and tumor cells was used to control the morphology and size of Fe_3_O_4_ in process of biomimetic mineralization. Ginger extract was used as antioxidant and chelator to collaborate in the biomimetic mineralization process. The obtained Fe_3_O_4_-QY-G nanoparticles are of superparamagnetism and presented good MRI imaging efficiency. Cell viability and targeting tests showed Fe_3_O_4_-QY-G nanoparticles can not only suppress the growth of A2780 cells but also target to them. In conclusion, the Fe_3_O_4_-QY-G nanoparticles may have potential application in targeting tumor therapy or angiography.

## Supplementary Materials

Table S1: Side chain functional groups contained in 14-mer peptide. Figure S1: (a) XRD pattern of Fe_3_O_4_-TVN and Fe_3_O_4_-SVE nanoparticles, (b) TEM image of Fe_3_O_4_-TVN nanoparticles, (c) TEM image of Fe_3_O_4_-SVE nanoparticles. Figure S2: TEM images of Fe_3_O_4_ nanoparticles prepared by adding different concentrations of ginger extract: (a) 3 mL, (b) 2 mL, (c) 1 mL, (d) 0.5 mL. Figure S3. TG and DSC curves of (a) Fe_3_O_4_ nanoparticles (b) Fe_3_O_4_-QY nanoparticles (c) Fe_3_O_4_-QY-G nanoparticles. Figure S4. TEM images of Fe_3_O_4_ nanoparticles prepared by adding different amount of peptide (a) 30 mg, (b) 40 mg, (c) 50 mg, (d) 60 mg; (e) XRD pattern of Fe_3_O_4_ nanoparticles prepared by adding different amount of peptide.
