# Supplementary material for: Biomimetic Mineralization of Magnetic Iron Oxide Nanoparticles Mediated by Bi-Functional Copolypeptides
Source: Molecules. 2019 Apr 10;24(7):1401. doi: 10.3390/molecules24071401 (PMC6480056; doi:10.3390/molecules24071401)
Supplement: Supplementary file 1 [file molecules-24-01401-s001.pdf]

Supporting

Table S1. Side chain functional groups contained in 14-mer peptide

	Q	Q	T	N	W	S	L	T	V	N	F	K	L	Y
C=O	Δ	Δ		Δ						Δ				
-OH			Δ			Δ		Δ						Δ
-COOH	Δ													
Pos												Δ		
Hyl	Δ	Δ	Δ	Δ		Δ		Δ		Δ		Δ		Δ

-OH：Hydroxyl groups, -COOH: Carboxyl groups, C=O: Carbonyl group,
Pos: Positive charge groups, Hyl: Hydrophilic groups

1. Verification of peptide (TVNFKLY)' Fe_3O_4-MNPs controlling capability

According to the method described in 4.2, Fe_3O_4 was generated by using heptapeptide with Fe_3O_4 affinity (TVNFKLY, TVN) and random heptapeptide (SVEGHDP, SVE). The results are as follows:

The samples Fe_3O_4-TVN indicated the diffraction peaks of Fe_3O_4 crystals (JCPDS No. 89-3854), centered at around 30.02° (200), 35.7° (311), 43.27° (400), 57.14° (511), and 62.70° (440). which showed that the formed composite was cubic spinel Fe_3O_4. The patterns of sample Fe_3O_4-SVE were similar to those of Fe_3O_4-TVN. However, the relative intensity of the specific peak for Fe_3O_4 decreased. Therefore, it is reasonable to believe that Fe_3O_4 with better crystallinity structure was mineralized via using TVN as a template. Figure S1 (b) and (c) indicated that the Fe_3O_4-TVN nanoparticles presented better dispersibility and more uniform size than Fe_3O_4-SVE, which proved that TVN could ~~mediated~~ mediate the formation of Fe_3O_4.

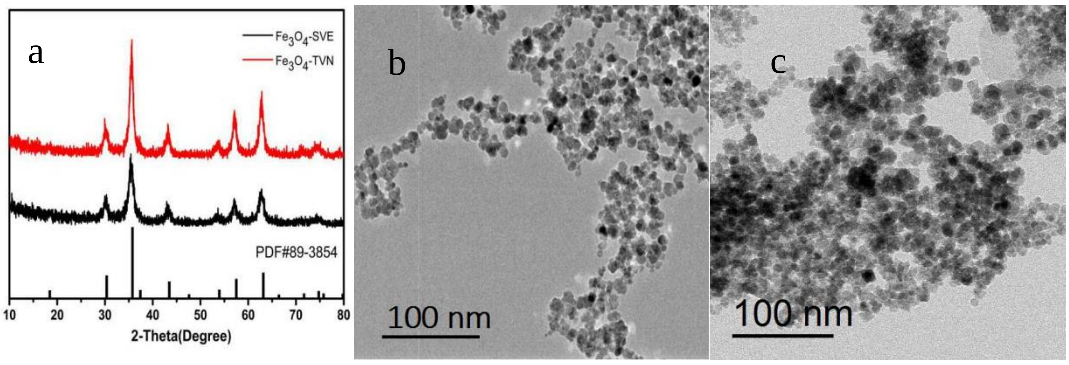

Figure S1. (a) XRD pattern of Fe_3O_4-TVN and Fe_3O_4-SVE nanoparticles, (b) TEM image of Fe_3O_4-TVN nanoparticles, (c) TEM image of Fe_3O_4-SVE nanoparticles.

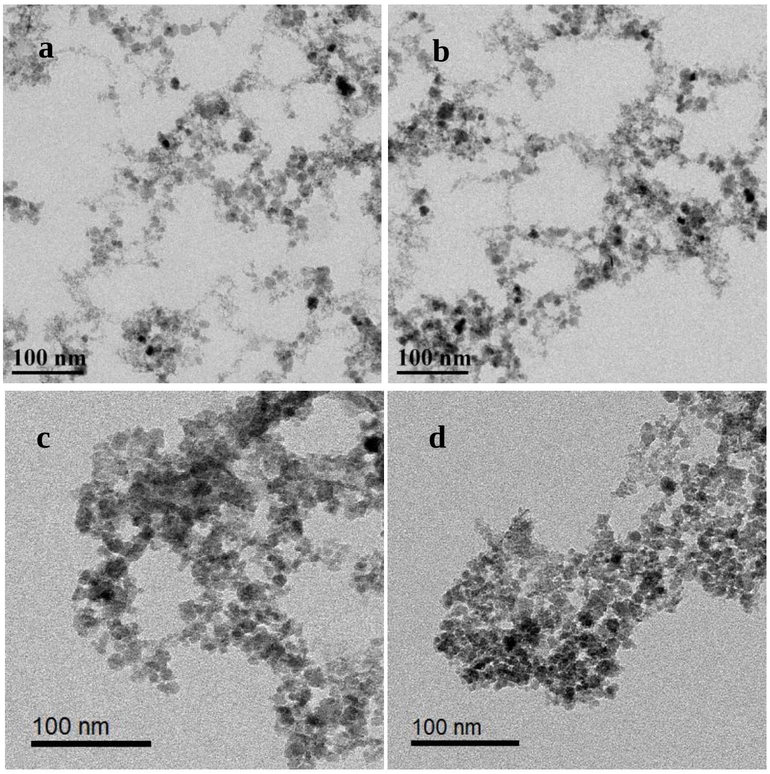

Figure S2. TEM images of Fe_3O_4 nanoparticles prepared by adding different concentrations of ginger extract: (a) 3 ml, (b) 2 ml, (c) 1 ml, (d) 0.5 ml.

2. Results of TG --DSC

The results of thermo-gravimetric (TG) and differential scanning calorimeter (DSC) are shown in Figure S3. In comparison, the weight loss of sample prepared by coprecipitation method (a) was about 4.61%. The weight losses at the temperatures ranging from 35 °C to about 100 °C, and from 200 °C to 360 °C were due to desorption of physically and chemically adsorbed water, respectively. The exothermic peak at 271.9°C was related to the transformation of Fe_3O_4 to γ-Fe_2O_3 [1]. The exothermic peak at 504.7°C corresponded to phase change and was associated to formation of ε-Fe_2O_3. The Fe_3O_4-QY (b), Fe_3O_4-QY-G (c) nanoparticles sample also showed the endothermic peaks around the above temperature but the mass loss was 8,73% and 13.82% respectively in the range of 35°C to 360°C. In addition to the desorption of physically and chemically adsorbed water mentioned above, the weight loss of sample Fe_3O_4-QY-G was also attributable to the decomposition and vaporization of organic compounds contained in the peptides and ginger extracts [2-4].

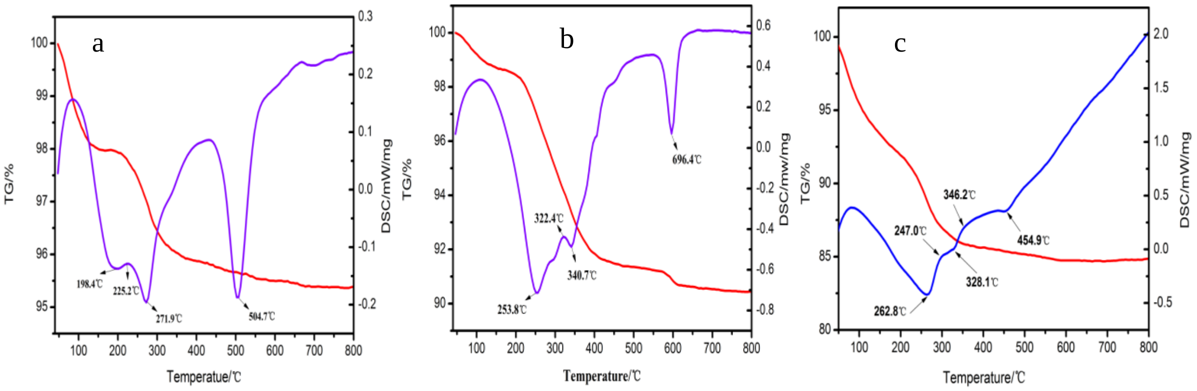

Figure S3. TG and DSC curves of (a) Fe_3O_4 nanoparticles (b) Fe_3O_4-QY nanoparticles (c) Fe_3O_4-QY-G nanoparticles.

3. Results of Fe_3O_4 nanoparticles prepared by adding different concentrations of peptide (QY)

As seen in Figure S4 (a-d), at lower amount of addition (30，40 mg per 50 mL), the ideal particle size and distribution were not obtained. However, when the addition amount reached 60 mg (per 50 mL), the particle size became smaller with the serious aggregation. On the one hand, in the absence of further modification, as the particle size decreases, the larger the specific surface area is, the higher the concentration of atoms on the particle surface is, which will lead to the lower coordination number of atoms on the surface and higher surface energy. On the other hand,when there were too many peptide molecules on the surface of Fe_3O_4, the particles may aggregate due to the entanglement of the peptide. Both of the above reasons may result in more serious particle agglomeration. Therefore, we chose 50 mg (per 50 mL) as the additive amount. In addition, as seen from the XRD pattern (Figure S4 (e)), the nanoparticles obtained were all the Fe_3O_4 with cubic inverse spinel structure. It means that the addition of peptide have no significant influence on the crystal structure of Fe_3O_4

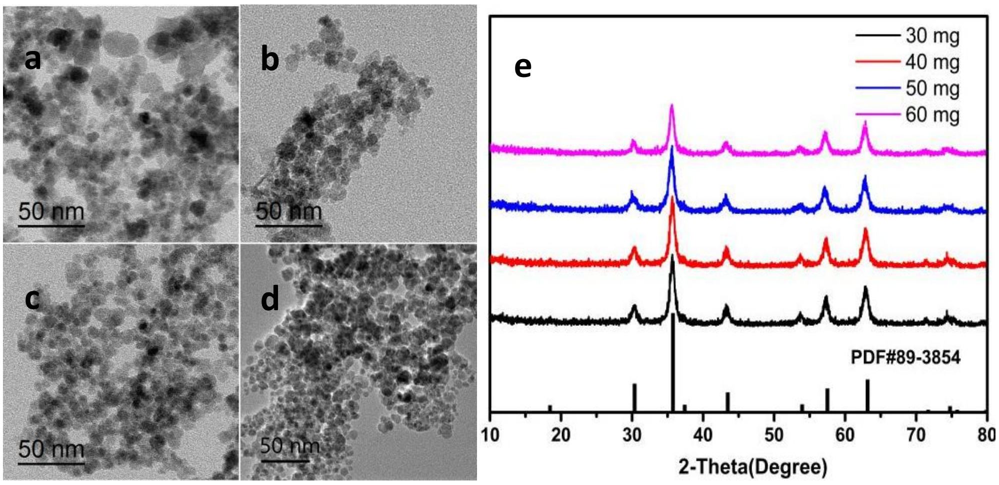

Figure S4. TEM images of Fe_3O_4 nanoparticles prepared by adding different amount of peptide (a) 30 mg, (b) 40 mg, (c) 50 mg, (d) 60 mg; (e) XRD pattern of Fe_3O_4 nanoparticles prepared by adding different amount of peptide.

References

1. Mody, V.V; Siwale, R; Singh, A; Mody, H.R. Introduction to metallic nanoparticles. *J Pharm Bioallied Sci*, **2010**, *2*, 282-289.
2. Mungi, S; Chi-Tang, H. Ammonia Generation during Thermal Degradation of Amino Acids. *J Agr Food Chem*, **1995**, *43*, 3001-3003.
3. Franco, B; Zhang, SF; Sujit, K.K; Lu, L. Mass Spectrometry Characterization of the Thermal Decomposition/Digestion (TDD) at Cysteine in Peptides and Proteins in the Condensed Phase. *J Am Soc Mass Spectr*, **2011**, *22*, 1926-1940.
4. Zhang, L.M; Xie, W.G; Zhao, X; Liu, Y; Gao, W.Y. Study on the morphology, crystalline structure and thermal properties of yellow ginger starch acetates with different degrees of substitution. *Thermochim Acta*, **2009**, *495*, 57-62.
